# Comparative effectiveness research trial for antidepressant incomplete and non-responders with treatment resistant depression (ASCERTAIN-TRD) a randomized clinical trial

**DOI:** 10.1038/s41380-024-02468-x

**Published:** 2024-03-07

**Authors:** George I. Papakostas, Madhukar H. Trivedi, Richard C. Shelton, Dan V. Iosifescu, Michael E. Thase, Manish K. Jha, Sanjay J. Mathew, Charles DeBattista, Mehmet E. Dokucu, Olga Brawman-Mintzer, Glenn W. Currier, William Vaughn McCall, Mandana Modirrousta, Matthew Macaluso, Alexander Bystritsky, Fidel Vila Rodriguez, Erik B. Nelson, Albert S. Yeung, Anna Feeney, Leslie C. MacGregor, Thomas Carmody, Maurizio Fava

**Affiliations:** 1 Massachusetts General Hospital and Harvard Medical School, Boston, MA USA; 2 University of Texas Southwestern Medical Center, Dallas, TX USA; 3 University of Alabama at Birmingham, Birmingham, AL USA; 4 Nathan Kline Institute for Psychiatric Research and New York University School of Medicine, New York, NY USA; 5 Perelman School of Medicine of the University of Pennsylvania, Philadelphia, PA USA; 6 Baylor College of Medicine, Houston, TX USA; 7 Stanford University School of Medicine, Stanford, CA USA; 8 Dartmouth Geisel School of Medicine, Lebanon, NH USA; 9 Medical University of South Carolina, Charleston, SC USA; 10 Morsani College of Medicine, University of South Florida, Tampa, FL USA; 11 Medical College of Georgia, Augusta, GA USA; 12 University of Manitoba, Winnipeg, MB Canada; 13 University of Kansas School of Medicine, Wichita, KS USA; 14 Semel Institute for Neuroscience and Human Behavior, University of California, Los Angeles, LA USA; 15 University of British Columbia, Vancouver, BC Canada; 16 University of Cincinnati Academic Health Center, Cincinnati, OH USA

**Keywords:** Depression, Drug discovery

## Abstract

Further research is needed to help improve both the standard of care and the outcome for patients with treatment-resistant depression. A particularly critical evidence gap exists with respect to whether pharmacological or non-pharmacological augmentation is superior to antidepressant switch, or vice-versa. The objective of this study was to compare the effectiveness of augmentation with aripiprazole or repetitive transcranial magnetic stimulation versus switching to the antidepressant venlafaxine XR (or duloxetine for those not eligible to receive venlafaxine) for treatment-resistant depression. In this multi-site, 8-week, randomized, open-label study, 278 subjects (196 females and 82 males, mean age 45.6 years (SD 15.3)) with treatment-resistant depression were assigned in a 1:1:1 fashion to treatment with either of these three interventions; 235 subjects completed the study. 260 randomized subjects with at least one post-baseline Montgomery-Asberg Depression Rating (MADRS) assessment were included in the analysis. Repetitive transcranial magnetic stimulation (score change (standard error (se)) = −17.39 (1.3) (*p* = 0.015) but not aripiprazole augmentation (score change (se) = −14.9 (1.1) (*p* = 0.069) was superior to switch (score change (se) = −13.22 (1.1)) on the MADRS. Aripiprazole (mean change (se) = −37.79 (2.9) (*p* = 0.003) but not repetitive transcranial magnetic stimulation augmentation (mean change (se) = −42.96 (3.6) (*p* = 0.031) was superior to switch (mean change (se) = −34.45 (3.0)) on the symptoms of depression questionnaire. Repetitive transcranial magnetic stimulation augmentation was shown to be more effective than switching antidepressants in treatment-resistant depression on the study primary measure. In light of these findings, clinicians should consider repetitive transcranial magnetic stimulation augmentation early-on for treatment-resistant depression.

**Trial registration:** ClinicalTrials.gov, NCT02977299

## Introduction

MDD is a serious, debilitating, life-shortening illness that affects many persons of all ages and backgrounds. A particularly critical decision in everyday practice is choosing what to do next when patients with MDD present after antidepressant treatments have failed to produce a clinical response, and a large evidence gap exists with respect to this common clinical scenario [1–8]. There are many augmentation and switch options to choose from for patients with TRD, each of these with varying degrees of evidence [9, 10]. As discussed in a review on the integrative management of TRD [11], the authors conclude that two pharmacotherapeutic options have accrued the most evidence for efficacy from well-controlled studies of TRD patients: switching to an antidepressant of a different class and augmenting the antidepressant with atypical antipsychotics. Despite both augmentation with atypical antipsychotic agents and switching to a different antidepressant class having well-established efficacy, strikingly, there has never been a direct comparison of them for TRD [11] .

To date, aside from the atypical antipsychotic agents, electroconvulsive therapy (ECT) and esketamine, repetitive transcranial magnetic stimulation (rTMS) represents the only other modality approved for use in MDD patients who have not responded to antidepressant therapy [12, 13]. rTMS has been extensively studied for MDD (29 randomized clinical trials (RCTs) [14]), as well as in TRD specifically (18 RCTs [14]), including as augmentation, with an evidence-base greater than for almost any other intervention for TRD save for the atypical antipsychotic agents (21 RCTs [14]). The results of a meta-analysis showed rTMS to be efficacious in MDD and in TRD, with equivalent efficacy when delivered as monotherapy or augmentation [15]. There is also accumulating evidence for the durability of the antidepressant effect of rTMS in clinical practice [16].

In summary, TRD remains a significant challenge for clinicians and patients alike, and further research is needed to help improve both the standard of care and the outcome for patients with TRD. A critical evidence gap exists with respect to whether pharmacological or non-pharmacological augmentation is superior to antidepressant switch, or vice-versa. An additional evidence gap exists with regards to our limited knowledge as to which US Food and Drug Administration (FDA)-approved augmentation versus switch strategies to employ. Our proposal was designed to address these evidence gaps by comparing two FDA-approved treatments for patients with TRD, namely augmentation with atypical antipsychotics versus augmentation with repetitive transcranial magnetic stimulation (rTMS), with one of the commonly used strategies for TRD: switching to the serotonin-norepinephrine reuptake inhibitors (SNRI) venlafaxine extended release (XR) or duloxetine.

## Methods

This was a multi-site, eight-week, randomized rater-blinded trial comparing three treatment arms for MDD patients with TRD who are currently on ongoing, stable and adequate antidepressant therapy (ADT): a) aripiprazole augmentation, b) rTMS augmentation, and c) switching to venlafaxine XR (or duloxetine for patients who had received venlafaxine during their current major depressive episode) (clinicaltrials/gov: NCT02977299). This trial was conducted according to the U.S. FDA guidelines and the Declaration of Helsinki. IRB-approved written informed consent was obtained from all patients before any protocol-specified procedures were carried out (IRB approval was site specific). Subjects were enrolled across 17 sites in the United States and Canada, where treatment assignment was performed and patients followed clinically.

### Key inclusion and exclusion criteria

A subject was considered to be eligible for inclusion only if all inclusion criteria were met. Subjects were a) women and men ages 18–80, b) with MDD, of at least 12 weeks duration, c) who had a Montgomery-Asberg Depression Rating Scale (MADRS [17]) score of at least 20 at screening and baseline, and d) who met criteria for TRD during the current major depressive episode, documented in the MGH Antidepressant Treatment History Questionnaire (ATRQ) [18]. TRD was defined as being non-responders during the current episode (less than 50% of symptom improvement) to two or more depression treatment trials of adequate dose and duration, as defined by the MGH ATRQ. In addition, included subjects had documented non-response to their current antidepressant. Patients found eligible during site screening were scheduled for a remote assessment by clinicians at MGH CTNI for confirmation of study eligibility. Additional detail regarding inclusion and exclusion criteria is included in the Supplementary Material (Supplementary Methods 1a).

### Randomized phase

At baseline, MGH CTNI as well as site clinicians administered the MADRS, and eligible patients were randomized 1:1:1 in an open-label fashion to one of the three study interventions. Randomization was performed within site with a computer number generating sequence conducted at the study data coordinating center, and using randomly varied blocks of 3, 6, or 9. Post-baseline visits occurred at weeks 1, 2, 3, 4, 6 and 8. At the beginning of each post-baseline visit, MADRS was performed by an MGH CTNI rater blinded to treatment assignment, and the score was then provided to the site clinician who would record side-effects and adjust medications as needed per the guidelines below. Site clinicians did not complete the MADRS post-baseline as they were unblinded as to treatment assignment. The self-rated symptoms of depression questionnaire (SDQ [19]) was administered at each site visit. Additional detail regarding each of the treatment arms is included in the supplementary material (Supplementary Methods 1b). Serious adverse events were defined as per the FDA (https://www.fda.gov/safety/reporting-serious-problems-fda/what-serious-adverse-event).

### Statistical analysis

The study primary outcome was defined as the change in MADRS scores (MGH CTNI-administered). All efficacy analyses were conducted on the modified intent-to-treat dataset (MITT), where all patients with any post-baseline data were included. All tests were two-sided. Because two different augmentation arms were each compared with switching, we used a Bonferroni corrected alpha of 0.025, and no interim analyses were planned or conducted. Since the objective of the original request for proposals by PCORI was to compare augmentation versus switching, no formal planning for the comparison between the augmentation groups was made, therefore justifying an alpha of 0.025 rather than 0.016. Analyses were conducted using SAS V9.4. For the MADRS analyses, mixed-effects models with repeated measures (MMRM) were conducted with treatment group (augment versus switch) as the between-subjects factor, time as the within-subjects factor, and a group by time interaction term. The baseline measurement of the MADRS was included as a covariate and not as a predictor. Site and site by treatment group interactions were examined and retained in the model only if a significant improvement in model fit (based on the Schwarz’s Bayesian Criterion) resulted. The linearity assumption was examined by a change-over-time graph by subject and by testing if the model goodness of fit could be significantly increased by inclusion of a time-squared term or the transformation ln (week+1). The assumptions of normality and homogeneity of variance and the presence of outliers were examined by reference to the distribution of model residuals. Determination of a significant treatment effect was based on the model treatment group effect or treatment group by time interaction [20]. The self-rated SDQ was analyzed in an identical manner to the MADRS. SAS Proc Mixed was used to perform the analyses. Sensitivity analyses were performed for the MADRS to examine the effect of excluding participants randomized to venlafaxine/duloxetine with rTMS randomizations were paused due to COVID.

Under the MMRM approach, assuming a small-to-medium effect size of f = 0.12 (based on Cohen’s f for analysis of variance where f = 0.10 is a small effect [21]) and a total of seven remote assessment observations (baseline, and visits 1–6), each treatment arm will require *N* = 170, for a total sample size of 510 patients powered at 80%. Since randomized patients with no post-baseline severity measurements were not included in the analysis, we assumed a loss of 20%. Therefore, the sample size was increased by 20% to 639 with the goal of obtaining 510 randomized subjects with one post-baseline assessment.

Logistic regression models were fit with MADRS remission (an exit MADRS of 10 or less) and response (50% or more baseline to exit improvement) as the dependent variables, and terms for treatment group and baseline MADRS and as covariates along with site as a covariate if needed. Rates and NNT were reported. NNT for a binary outcome for treatments A versus B represents the number of participants who must be treated with A in order to have one more response/remission than if the same number of participants were treated with B.

## Results

**Fig. 1:**
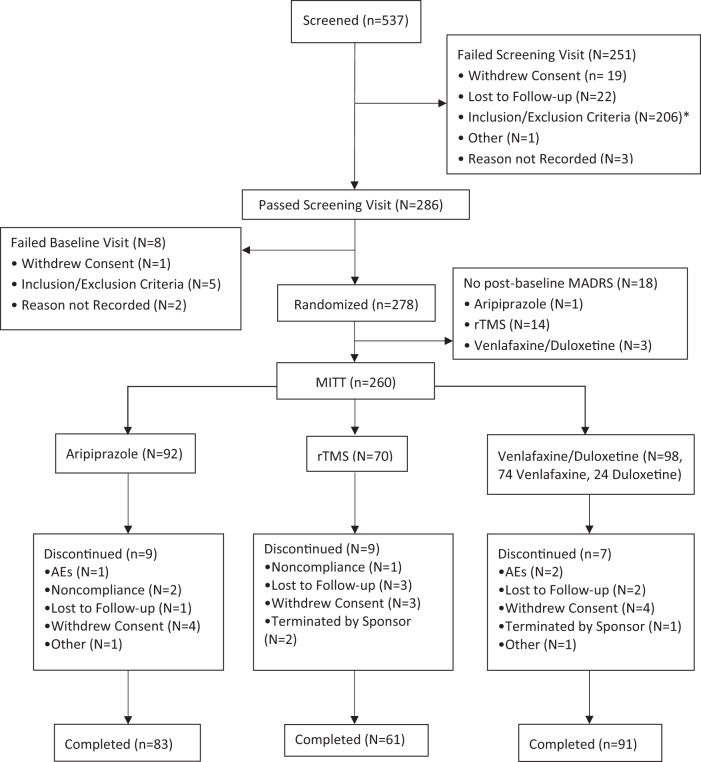
CONSORT flow diagram. AE Adverse events, MITT Modified intent-to-treat, MADRS Montgomery Asberg Depression Rating Scale, rTMS repetitive transcranial magnetic stimulation. *Five most frequently cited reasons for not meeting Inclusion/Exclusion Criteria: Criteria for treatment resistant depression not met during current episode (*n* = 54, 26.2%) Did not pass CNTI remote assessment (*n* = 45, 21.8%). Criteria for current antidepressant not met (*n* = 30, 14.6%). Current episode less than 12 weeks duration (*n* = 21, 10.2%). History of bipolar disorder or psychosis (*n* = 20, 9.7%).

A total of 278 eligible participants were randomized to treatment during the course of the trial. This was far less than the projected 639. Main reasons for this included a delay in study startup, slow recruitment (aripiprazole and venlafaxine XR/duloxetine were chosen due to their popularity so as to render study results more generalizable, but this also meant that many potential subjects had already received treatment with these two agents during their current depressive episode), as well as the COVID pandemic. Of these 278 subjects, 260 (95.2%) had at least one post-baseline MADRS score and were eligible for the MITT analysis of the primary outcome. Participants were enrolled from 07/13/2017 to 12/22/2021, with the study allowed to continue for the initially planned duration (no early stop). Altogether, 235 (90.3%) of 260 subjects in the MITT analysis completed the 8-week randomized trial. Disposition of study participants is shown in Fig. 1, and baseline clinical and demographic factors of randomized participants shown in Table 1. Baseline antidepressants by group are shown in Supplementary Table 1. See Supplementary Material (Supplementary Tables 2–6) for reports of adverse events and serious adverse events according to the treatment groups. There were no drug or rTMS-related serious adverse events during the course of the trial. Maximum doses were as follows: aripiprazole mean (SD) 9.0 (4.1) mg, median 10 mg, minimum 2 mg, maximum 20 mg, venlafaxine mean (SD) 190.7 (66.7) mg, median 225 mg, minimum 38 mg, maximum 375 mg, duloxetine mean (SD) 97.5 (25.4) mg, median 105 mg, minimum 60 mg, maximum 120 mg. Only one subject required treatment with benztropine for aripiprazole-related akathisia.

**Table 1:** Baseline demographic and clinical factors of randomized participants.

Variable	All *N* (%)	Aripiprazole augmentation *N* (%)	rTMS augmentation *N* (%)	Venlafaxine XR/Duloxetine Switch *N* (%)
Female	196 (70.5)	69 (74.1)	58 (69.0)	69 (68.3)
White	203 (74.3)	66 (72.5)	65 (80.2)	72 (71.2)
African American	41 (15.0)	15 (16.4)	7 (8.6)	19 (18.8)
Race/Other	29 (10.6)	10 (10.9)	9 (11.1)	10 (9.9)
Hispanic ethnicity	29 (10.5)	9 (9.6)	6 (7.1)	14 (14.2)
**Variable**	**All mean (SD)**	**Aripiprazole augmentation mean (SD)**	**TMS augmentation mean (SD)**	**Venlafaxine XR/Duloxetine switch mean (SD)**
Age (years)	45.6 (15.3)	47.0 (16.1)	43.8 (14.5)	45.6 (15.3)
Number of failed trials	2.85 (1.0) Median = 3 25th percentile = 2 75th percentile = 3 Minimum = 2 Maximum = 8	2.94 (1.1) Median = 3 25th percentile = 2 75th percentile = 3 Minimum = 2 Maximum = 8	2.82 (1.0) Median = 2.5 25th percentile = 2 75th percentile = 3 Minimum = 2 Maximum = 6	2.80 (0.9) Median = 3 25th percentile = 2 75th percentile = 3 Minimum = 2 Maximum = 6
MADRS total score	32.6 (6.3) Median = 32 25th percentile = 28 75th percentile = 37 Minimum = 20 Maximum = 46	33.1 (6.0) Median = 33 25th percentile = 29 75th percentile = 37 Minimum = 20 Maximum = 46	33.1 (6.0) Median = 33 25th percentile = 29 75th percentile = 38 Minimum = 21 Maximum = 45	33.0 (6.0) Median = 32 25th percentile = 28 75th percentile = 38 Minimum = 21 Maximum = 46
SDQ total score	156.1 (25.4) Median = 154 25th percentile = 138 75th percentile = 174	155.2 (22.8) Median = 155 25th percentile = 138 75th percentile = 169	155.9 (26.1) Median = 155 25th percentile = 138 75th percentile = 175	156.9 (27.1) Median = 153 25th percentile = 140 75th percentile = 177
	Minimum = 95 Maximum = 223	Minimum = 113 Maximum = 205	Minimum = 95 Maximum = 222	Minimum = 96 Maximum = 223

*MADRS* Montgomery Asberg Depression Rating Scale, *rTMS* Repetitive Transcranial Magnetic Stimulation, *SD* standard deviation, *SDQ* Symptoms of Depression Questionnaire.

One significant change in the protocol had to occur due to the COVID pandemic. Specifically, on 8/17/2020, an amendment previously approved by the sponsor was put into effect to continue enrolling in the medication treatment arms but not the rTMS arm (the study had been halted from 3/16/2020 due to COVID19). The rationale for this was to minimize the chances of COVID spreading to patients and staff, since rTMS required many in-person visits (please see rTMS methods section). All three arms of the study were re-initiated 3/1/2021. An analysis comparing rTMS to venlafaxine on the study primary outcome excluding venlafaxine subjects randomized during this period is reported in the supplemental section of this manuscript (Supplementary Table 7), and is in line with main results.

For all MMRM analyses, examination of model residuals indicated the residuals were normally distributed and no observations should be classified as outliers. For all models the spatial powers correlated errors covariance structure produced the best fitting model.

### Change in MADRS scores: aripiprazole augmentation versus switch to venlafaxine XR/duloxetine

**Fig. 2:**
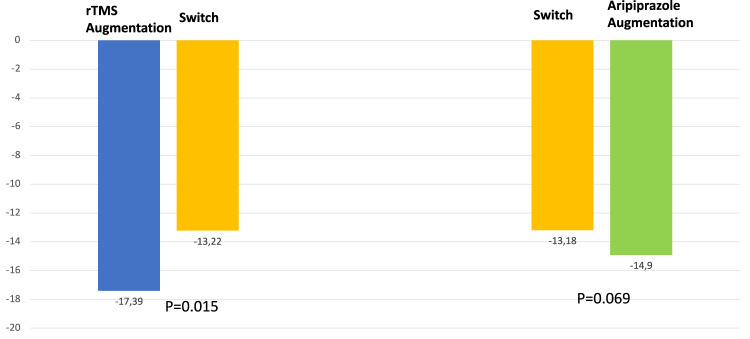
Model adjusted change in MADRS scores. MADRS Montgomery Asberg Depression Rating Scale, rTMS Repetitive Transcranial Magnetic Stimulation. Alpha = 0.025.

A plot of change over time for each participant showed the change was non-linear. Examination of the goodness of fit statistic indicated that use of the log(time+1) transformation produced the best fitting model. Neither the treatment group main effect (*p* = 0.069) nor week by treatment group interaction effect (*p* = 0.708) were significant. Model estimated mean (SE) change from baseline to week 8 in MADRS scores for aripiprazole augmentation versus switching to venlafaxine XR/duloxetine were −14.9 (1.1) versus −13.18 (1.1). Mean (SE) slopes for MADRS scores for aripiprazole augmentation versus switching to venlafaxine XR/duloxetine were −5.85 (1.1) versus −6.26 (0.8). A graphic depiction of the outcome is shown in Fig. 2.

### Change in MADRS scores: rTMS augmentation versus switch to venlafaxine XR/duloxetine

A plot of change over time for each participant showed a non-linear change. Examination of the goodness of fit statistic indicated that use of the log(time+1) transformation produced the best fitting model. The treatment group interaction effect was not significant (*p* = 0.234), but the week by treatment group effect was significant at the pre-specified level of alpha=0.025 (*p* = 0.015). Model estimated mean (SE) level for MADRS scores for rTMS augmentation versus switching to venlafaxine XR/duloxetine were −17.39 (1.3) versus −13.22 (1.1). Mean (SE) slopes for MADRS scores for rTMS augmentation versus switching to venlafaxine XR/duloxetine were −8.95 (1.1) versus −6.26 (0.7). A graphic depiction of the outcome is shown in Fig. 2.

### Change in SDQ scores: Aripiprazole augmentation versus switch to venlafaxine XR/duloxetine

**Fig. 3:**
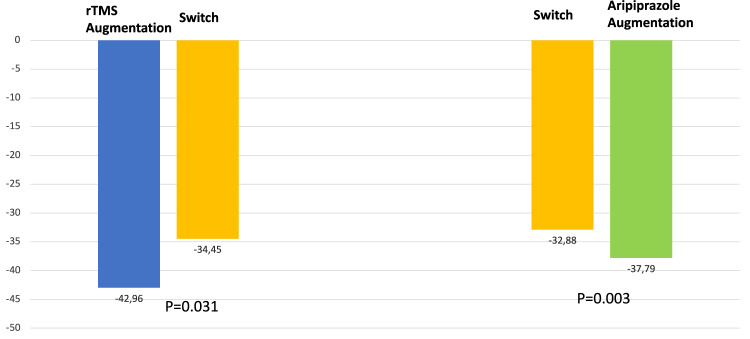
Model adjusted change in SDQ scores. SDQ Symptoms of Depression Questionnaire, rTMS Repetitive Transcranial Magnetic Stimulation. Alpha = 0.025.

A plot of change over time for each participant showed a deviation from linearity. Examination of the goodness of fit statistic indicated that use of log(time+1) transformation produced the best fitting model. The treatment group main effect was significant at the pre-specified level of alpha=0.025 (*p* = 0.003), while the week by treatment group interaction effect was not significant (*p* = 0.172). Model estimated mean (SE) change in SDQ scores for aripiprazole augmentation versus switching to venlafaxine XR/duloxetine were −37.79 (2.9) versus −32.88 (2.8). Mean (SE) slopes for SDQ scores for aripiprazole augmentation versus switching to venlafaxine XR/duloxetine were −9.80 (3.1) versus −14.07 (2.2). A graphic depiction of the outcome is shown in Fig. 3.

### Change in SDQ scores: rTMS augmentation versus switch to venlafaxine XR/duloxetine

A plot of change over time for each participant showed a slight deviation from linearity. Examination of the goodness of fit statistic indicated that use of time without transformation produced the best fitting model. The treatment group main effect (*p* = 0.031) and the week by treatment group interaction effect (*p* = 0.832) were not significant. Model estimated mean (SE) change in SDQ scores for rTMS augmentation versus switching to venlafaxine XR/duloxetine were −42.96 (3.6) versus −34.45 (3.0). Mean (SE) slopes for SDQ scores for rTMS augmentation versus switching to venlafaxine XR/duloxetine were −3.07 (0.7) versus −2.93 (0.4). A graphic depiction of the outcome is shown in Fig. 3.

### MADRS response and remission

**Fig. 4:**
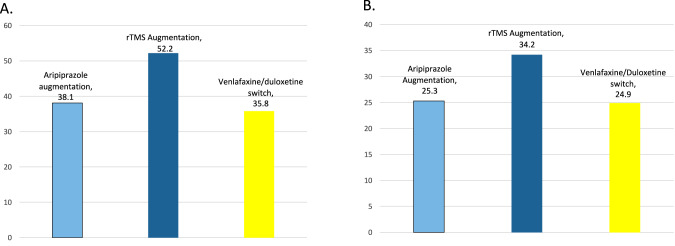
MADRS response and remission rates comparing different study arms. **A** MADRS response rates (%); **B** MADRS remission rates (%). MADRS Montgomery-Asberg Depression Rating Scale (MADRS), rTMS Repetitive Transcranial Magnetic Stimulation; y-axis = % response/remission, x-axis = study arms.

For all logistic regression models, it was found that site effects did not significantly improve the fit of the model and so were removed from the model leaving treatment group and baseline MADRS as predictors. MADRS response (50% or greater reduction in symptoms from baseline to exit visit) and remission (total score less than 10 at the exit visit) was not significantly different between aripiprazole augmentation versus venlafaxine XR/duloxetine switch subjects (model estimated response rate = 0.381 and 0.358, number needed to treat (NNT) = 44, *p* = 0.743; model estimated remission rate = 0.253 and 0.249, NNT = 250.0, *p* = 0.946, respectively). MADRS response and remission was not significantly different at the pre-specified alpha = 0.025 level between rTMS augmentation versus venlafaxine XR/duloxetine switch subjects (model estimated response rate = 0.522 and 0.358, NNT = 7, *p* = 0.038; model estimated remission rate = 0.342 and 0.249, NNT = 11, *p* = 0.203, respectively). A graphic depiction of these outcomes is shown in Fig. 4.

## Discussion

The present study is the first randomized effectiveness study to compare augmentation versus switching in a general outpatient population with TRD. Results of our study show a greater reduction in depressive symptoms following rTMS augmentation than switching to venlafaxine XR/duloxetine. The magnitude of the difference in efficacy expressed in mean MADRS reduction was 4.17 points. Although our study was not designed or powered to detect a statistically significant difference in response or remission rates between the two groups, the NNT for response and remission was, approximately, 7 and 11 respectively. Taken together, these results are informative for clinical practice in TRD, and support rTMS augmentation over switching for this patient population. Future studies comparing rTMS augmentation with other treatment strategies specifically for MDD patient populations not included in our trial (i.e. adolescents, elderly) are warranted. Finally, it is worth noting emphasizing in the discussion that the superiority of adjunctive rTMS to an antidepressant switch in TRD patients contrasts with the comparatively more modest efficacy of rTMS monotherapy for TRD as shown in the meta-analysis of 24 RCTs by Lam et al. [22] (pooled response of 25%, pooled remission of 9%). The present findings are consistent with the literature suggesting a larger effect with rTMS as adjunctive (rather than mono-) therapy (e.g., as suggested by the meta-analysis of 7 RCTs as augmentation which found a pooled response rate of 46%, an OR of 5.12 and an SMD of 0.86 [23].

Our study was also the first to compare augmenting with an atypical antipsychotic agent versus switching to venlafaxine/duloxetine in TRD. In contrast to the VA Augmentation and Switching Treatments for Improving Depression Outcomes (VAST-D) study [24] no statistically significant difference in efficacy between these two treatments was found on the primary outcome measure (MADRS, *p* = 0.069). However, similar to VAST-D, aripiprazole augmentation was found superior to switching to venlafaxine XR/duloxetine on a patient-rated scale (SDQ). Therefore, it is quite possible that a significant, treatment effect could have been detected on the MADRS, as was in the SDQ, if we had been able to enroll the 639 participants indicated by the power analysis in ASCERTAIN (which involved a sample size just 16% of that of VAST-D). Differences in study design such as population (predominant male veteran-based versus general adult outpatient), disease stage (failure of at least one antidepressant versus TRD), or switch agent (bupropion versus venlafaxine/duloxetine) may have also contributed to differences in study results. In light of the extensive literature focusing on the use of aripiprazole augmentation in MDD, and advantage over switching on patient-rated symptoms, our study lends further support regarding the usefulness of this strategy for TRD.

In summary, despite the modest effect size favoring aripiprazole augmentation over switching in ASCERTAIN, and given clinical challenges and adverse outcomes associated with TRD, the sum of findings of ASCERTAIN and VAST-D continue to support the importance of augmenting with atypical antipsychotics versus switching for these patients. Rates of response (18.5%–46.6%) and remission (7.4%–36.8%) with aripiprazole augmentation in prior studies [25–29] are similar to those observed in this study (38.1% and 25.3%, respectively). Similarly, the rates of response (35.8%) and remission (24.9%) with switch to venlafaxine XR/duloxetine in ASCERTAIN is similar to those observed in the level 2 of Sequenced Treatment Alternatives to Relieve Depression [30], where individuals with MDD were switched to venlafaxine after inadequate improvement on treatment with citalopram (28.2% and 25.0%, respectively).

Several limitations of our study are worth mentioning. First, this was an open label trial where subjects were not blinded to treatment assignment. Blinding to treatment assignment when comparing augmentation versus switching is logistically very complicated, challenging and costly, since either matching drug-placebo pills must be created for multiple marketed antidepressants and dose levels or a lead-in with a single antidepressant must be employed which can increase sample size requirements by as much as four-fold [31] (Salloum et al., 2020). The same limitation applies to rTMS, which was not blinded to the patient, and to which the patient might attribute more expectancy as compared with a new drug. Instead, we chose to employ raters blinded to treatment assignment for the assessment of the study primary outcome measure. Second, we chose to limit drug treatment arms to a finite number of agents (aripiprazole, venlafaxine XR, duloxetine) as opposed to classes of agents. Whether our findings extend to other approved atypical antipsychotic agents (quetiapine, brexpiprazole, cariprazine) or antidepressants for MDD is a matter of speculation. The same argument can be made for ECT, ketamine, esketamine, or “accelerated” protocols of rTMS [32] which are gaining in popularity. Third, whether our findings extend to specific sub-populations that were excluded in ASCERTAIN, such as adolescents or the elderly, remains unknown. Future studies for this purpose are warranted. Fourth, the number of participants in the rTMS arm is lower as randomization to this arm was interrupted early in the COVID-19 pandemic due to restrictions imposed on the in-person visits necessary for this treatment arm. However, findings excluding venlafaxine subjects randomized during this period remain statistically significant for rTMS. Furthermore, a greater number of individuals in the rTMS arm did not have any post-baseline MADRS and may be related to unwillingness in some subpopulations to accept rTMS, which in turn may account for some differences in race and age distribution between the three groups. The limitation of the statistical method applied (which is standard in clinical trials in the field) is that differential early attrition can introduce bias. However, to include these subjects would mean imputing data, and imputed data is not real data. Nevertheless, we concede that our results apply more to subjects willing to commit to the requirements of rTMS. Finally, variations in treatment effect by site may be present but could not be adequately tested given the number of sites and the size of the study.

In conclusion, results of the current trial demonstrate the superiority of rTMS augmentation over switching to venlafaxine XR/duloxetine in TRD on clinician-rated symptoms of depression, with a moderate-to-large effect size. In addition, similar to VAST-D, a statistically significant advantage was found for aripiprazole augmentation versus switching on patient-rated symptoms of depression. These findings are informative for clinical practice in TRD and warrant future studies in select sub-populations such as adolescents or the elderly.

## Supplementary information

Supplementary material

## Data Availability

GIP and TC had full access to all the data in the study and takes responsibility for the integrity of the data and the accuracy of the data analysis. TC conducted and is responsible for the data analysis.
