# Supplementary material for: Comparative effectiveness research trial for antidepressant incomplete and non-responders with treatment resistant depression (ASCERTAIN-TRD) a randomized clinical trial
Source: Mol Psychiatry. 2024 Mar 7;29(8):2287–95. doi: 10.1038/s41380-024-02468-x (PMC11412904; doi:10.1038/s41380-024-02468-x)
Supplement: Supplementary file 1 — Supplementary material [file 41380_2024_2468_MOESM1_ESM.docx]

Supplementary information for

**Comparative Effectiveness Research Trial for Antidepressant Incomplete and Non-responders with Treatment Resistant Depression (ASCERTAIN-TRD)**

**A Randomized Clinical Trial**

George I Papakostas M.D., Madhukar H Trivedi M.D., Richard C Shelton M.D., Dan V Iosifescu M.D., Michael E Thase M.D., Manish K. Jha M.D., Sanjay J Mathew M.D., Charles DeBattista M.D., Mehmet E Dokucu M.D., Olga Brawman-Mintzer M.D., Glenn W Currier M.D., William Vaughn McCall M.D., Mandana Modirrousta M.D., Matthew Macaluso M.D., Alexander Bystritsky M.D., Fidel Vila Rodriguez M.D., Erik B Nelson M.D., Albert S. Yeung M.D., Anna Feeney M.D., Leslie C. MacGregor, Ph.D., Thomas Carmody Ph.D., Maurizio Fava M.D.

Corresponding author: George I Papakostas, MGH CTNI, One Bowdoin Square 9^th^ Floor, Boston MA 02114, [gpapakostas@partners.org](mailto:gpapakostas@partners.org). +16172904734

The PDF file includes:

1. **Supplementary Methods**
   1. Additional detail regarding inclusion and exclusion criteria
   2. Additional detail regarding treatment arms
2. **Supplementary Table 1**: Baseline Antidepressant
3. **Supplementary Table 2**: Treatment Emergent AEs or SAEs by Treatment Group
4. **Supplementary Table 3**: AEs for each Specific AE affecting 3% or more of participants
5. **Supplementary Table 4**: Treatment Emergent AEs and SAEs per Participant by Treatment Group
6. **Supplementary Table 5**: Number of Treatment Emergent AEs for each Specific AE affecting 3% or more of Participants
7. **Supplementary Table 6:** Highest Severity of AEs by Treatment Group among those with a Treatment Emergent AE
8. **Supplementary Table 7**: Sensitivity analysis - Primary analysis excluding participants randomized to Venlafaxine (N=20) while rTMS randomizations were paused due to COVID.
9. **Supplementary Methods**
10. Additional detail regarding inclusion and exclusion criteria

Inclusion and exclusion criteria

A subject was eligible for inclusion only if all inclusion criteria were met. Subjects were a) women and men ages 18-80, b) with Major depressive disorder (MDD), of at least 12 weeks duration, according to Diagnostic and Statistical Manual of Mental Disorders, Fifth Edition (DSM-5) criteria confirmed by the Mini International Neuropsychiatric Interview (MINI ^1^), c) who had a Montgomery-Asberg Depression Rating Scale (MADRS ^2^) score of at least 20 at screen and baseline, and d) who met criteria for TRD during the current major depressive episode documented in the MGH Antidepressant Treatment History Questionnaire (ATRQ) ^3^. TRD was defined as being non-responders during the current episode (less than 50% of symptom improvement) to two or more depression treatment trials of adequate dose and duration as defined by the MGH ATRQ. In addition, subjects e) were currently on an antidepressant of adequate dose (as defined by the MGH ATRQ) and duration (at least 8 weeks), with the antidepressant dose being stable over the past four weeks, and with documented (in the MGH ATRQ) non-response (less than 50% improvement) to the current antidepressant.

A potential participant was not eligible for participation in this study if any exclusion criterion was met. Reasons for exclusion were a) pregnant or breastfeeding women, or women of childbearing potential who are not using an accepted means of birth control, b) patients who had received treatment with repetitive transcranial magnetic stimulation (rTMS), atypical antipsychotic agents, electroconvulsive therapy (ECT), ketamine/ esketamine, or venlafaxine and duloxetine during the current episode, or lifetime vagus nerve stimulation (VNS), c) patients with any history of bipolar disorder or psychosis, or active alcohol or substance abuse disorders within the past 6 months, d) patients with suicidal ideation of the degree that, in the opinion of the evaluating clinician, participation in the study would place them at significantly increased risk of suicide, e) patients with unstable medical issues of such degree that, in the opinion of the evaluating clinician, participation in the study would place them at significant risk of a serious adverse event, f) patients who had not responded to more than five FDA-approved antidepressant treatment trials of adequate dose and duration during the current episode, or who did not respond to ECT in previous episodes, or g) patients on antipsychotic agents. Finally, patients on concomitant psychotropic agents (anticonvulsants, benzodiazepines, hypnotics, opiates, triiodothyronine (T3), modafinil, psychostimulants, buspirone, melatonin, omega-3 fatty acids, folate, l-methylfolate, s-adenosyl methionine, lithium) not on the same dose for at least four weeks prior to study entry or who do not agree to continue at the same dose during the acute phase of the study were excluded.

Remote assessment

Patients found eligible during site screening were scheduled for a remote assessment by clinicians at Massachusetts General Hospital (MGH) Clinical Trials Network and Institute (CTNI). During these remote assessments, inclusion criteria b, c, d, e and exclusion criteria b, f cited above were confirmed by re-administering the MINI (mood module only), MGH ATRQ and MADRS. Patients deemed eligible to continue in the study were then scheduled for their baseline visit.

1. Additional detail regarding treatment arms

Aripiprazole Augmentation: Patients randomized to this treatment arm were instructed to continue all permitted psychotropics at their current dose throughout the 8-week trial and initiate adjunctive aripiprazole. The starting dose was 5mg daily. The dose may have been reduced to as low as 2mg for tolerability issues. The dose could be adjusted in 2 or 5mg increments per visit. The minimum time per increment was 7 days. The maximum dose was set at 15mg daily. For patients who were not on potent cytochrome 2D6 inhibitors (such as paroxetine, fluoxetine, duloxetine) or on potent cytochrome 3A4 inhibitors (such as fluvoxamine and nefazodone) and who were able to tolerate 15mg daily, the maximum dose could be raised to 20mg daily for efficacy. As was performed in registrational studies ^4^ ^5^ ^6^ and to mimic clinical practice, propranolol (10-30mg once or twice daily) and/or benztropine (1-2 mg daily) could also be prescribed for akathisia, if needed.

Repetitive transcranial magnetic stimulation (rTMS): Patients randomized to this treatment arm were instructed to continue all permitted psychotropics at their current dose throughout the 8-week trial. Sites employed clinical rTMS stimulators with focal figure-of-eight coils, with a standard U.S. Food and Drug Administration (FDA)-approved protocol of 10Hz stimulation at 120% of the motor threshold intensity and a total of 3000 pulses per session targeting the left dorsolateral prefrontal cortex following standardized procedures (Beam F3 method ^7^). For patients who were unable to tolerate stimulation at 120% of motor threshold, clinicians administering the treatment had the flexibility to titrate intensity as needed for tolerability. This protocol complies with published safety guidelines ^8^. Sessions occurred daily Monday through Friday over 5 weeks. After this, there was a taper period of two weekly sessions during three additional weeks. This is as per the standard FDA-approved protocol ^9^.

Switching to Venlafaxine XR or Duloxetine: Patients randomized to this treatment arm were instructed to continue all permitted psychotropics throughout the 8-week trial, except for their antidepressant(s). They were instructed to discontinue all antidepressants and initiate venlafaxine or duloxetine that day, as direct switch to serotonergic antidepressants is well tolerated and avoids loss of precious therapeutic time ^10^, including to switching to venlafaxine in STAR*D ^11^. For patients who did not prefer a direct switch, or when clinically indicated otherwise in the opinion of the site investigator, a gradual tapering during the screening period was permitted, as long as a direct switch was made on the baseline visit from the final antidepressant dose. The starting dose of venlafaxine was 75mg daily (duloxetine 60mg). The dose of venlafaxine may have been reduced to as low as 37.5mg for tolerability issues (duloxetine 30mg). The maximum venlafaxine dose was set at 375mg as per day (duloxetine 120mg).

**References**

1. Sheehan DV, Lecrubier Y, Sheehan KH, et al. The Mini-International Neuropsychiatric Interview (M.I.N.I.): The Development and Validation of a Structured Diagnostic Psychiatric Interview for DSM-IV and ICD-10. *J Clin Psychiatry*. 1998;59(suppl 20):11980.

2. Montgomery SA, Asberg M. A new depression scale designed to be sensitive to change. *Br J Psychiatry*. 1979;134(4):382-389. doi:10.1192/bjp.134.4.382

3. Chandler GM, Iosifescu DV, Pollack MH, Targum SD, Fava M. Validation of the massachusetts general hospital Antidepressant Treatment History Questionnaire (ATRQ). *CNS Neurosci Ther*. 2010;16(5):322-325. doi:10.1111/j.1755-5949.2009.00102.x

4. Berman RM, Marcus RN, Swanink R, et al. The Efficacy and Safety of Aripiprazole as Adjunctive Therapy in Major Depressive Disorder: A Multicenter, Randomized, Double-Blind, Placebo-Controlled Study. *J Clin Psychiatry*. 2007;68(6):6122.

5. Berman RM, Fava M, Thase ME, et al. Aripiprazole augmentation in major depressive disorder: a double-blind, placebo-controlled study in patients with inadequate response to antidepressants. *CNS Spectr*. 2009;14(4):197-206. doi:10.1017/s1092852900020216

6. Marcus RN, McQuade RD, Carson WH, et al. The efficacy and safety of aripiprazole as adjunctive therapy in major depressive disorder: a second multicenter, randomized, double-blind, placebo-controlled study. *J Clin Psychopharmacol*. 2008;28(2):156-165. doi:10.1097/JCP.0b013e31816774f9

7. Beam W, Borckardt JJ, Reeves ST, George MS. An efficient and accurate new method for locating the F3 position for prefrontal TMS applications. *Brain Stimul Basic Transl Clin Res Neuromodulation*. 2009;2(1):50-54. doi:10.1016/j.brs.2008.09.006

8. Rossi S, Hallett M, Rossini PM, Pascual-Leone A. Safety, ethical considerations, and application guidelines for the use of transcranial magnetic stimulation in clinical practice and research. *Clin Neurophysiol*. 2009;120(12):2008-2039. doi:10.1016/j.clinph.2009.08.016

9. O’Reardon JP, Solvason HB, Janicak PG, et al. Efficacy and Safety of Transcranial Magnetic Stimulation in the Acute Treatment of Major Depression: A Multisite Randomized Controlled Trial. *Biol Psychiatry*. 2007;62(11):1208-1216. doi:10.1016/j.biopsych.2007.01.018

10. Montgomery SA, Nielsen RZ, Poulsen LH, Häggström L. A randomised, double-blind study in adults with major depressive disorder with an inadequate response to a single course of selective serotonin reuptake inhibitor or serotonin–noradrenaline reuptake inhibitor treatment switched to vortioxetine or agomelatine. *Hum Psychopharmacol Clin Exp*. 2014;29(5):470-482. doi:10.1002/hup.2424

11. Rush AJ, Trivedi MH, Wisniewski SR, et al. Bupropion-SR, Sertraline, or Venlafaxine-XR after Failure of SSRIs for Depression. *N Engl J Med*. 2006;354(12):1231-1242. doi:10.1056/NEJMoa052963

**Supplemental tables**

**Supplementary Table 1**: Current Antidepressant by Treatment Group

| **Current Antidepressant** | **Number All Participants** | **Number Ari Group** | **Number rTMS Group** | **Number Ven/Dul Group** |
| --- | --- | --- | --- | --- |
| Bupropion | 45 | 14 | 16 | 15 |
| Citalopram | 16 | 5 | 6 | 5 |
| Clomipramine | 1 | 0 | 1 | 0 |
| Desvenlafaxine | 8 | 1 | 5 | 2 |
| Duloxetine | 21 | 5 | 10 | 6 |
| Escitalopram | 49 | 17 | 12 | 20 |
| Fluoxetine | 40 | 14 | 10 | 16 |
| Levomilnacipran | 3 | 2 | 0 | 1 |
| Mirtazapine | 10 | 3 | 2 | 5 |
| Nortriptyline | 2 | 0 | 1 | 1 |
| Paroxetine | 5 | 0 | 1 | 4 |
| Sertraline | 46 | 18 | 7 | 21 |
| Venlafaxine | 20 | 10 | 6 | 4 |
| Vilazodone | 2 | 1 | 1 | 0 |
| Vortioxetine | 11 | 3 | 6 | 2 |

*Ari: aripiprazole, rTMS: repetitive transcranial magnetic stimulation, Ven/Dul: venlafaxine duloxetine*

**Supplementary Table 2**: Percent of Participants Experiencing a Treatment Emergent AE or SAE by Treatment Group

|  | **All Participants** | **Aripiprazole Group** | **rTMS Group** | **Venlafaxine Duloxetine**  **Group** | **p-value** |
| --- | --- | --- | --- | --- | --- |
| AEs | 48.9% (136/278) | 57.0% (53/93) | 39.3% (33/84) | 49.5% (50/101) | 0.062 |
| SAEs | 1.1% (3/278) | 0% (0/93) | 1.2% (1/84) | 2.0% (2/101) | 0.408 |

*AE: adverse events, SAE: serious adverse events, rTMS: repetitive transcranial magnetic stimulation.*

**Supplementary Table 3:** Percent of Participants Experiencing a Treatment Emergent AE for each Specific AE affecting 3% or more of participants

| **Adverse Event** | **All Participants N** | **All Participants %** | **Ari Group N** | **Ari Group %** | **rTMS Group N** | **rTMS Group %** | **Ven/ Dul Group N** | **Ven/ Dul Group**  **%** | **p-value** |
| --- | --- | --- | --- | --- | --- | --- | --- | --- | --- |
| Constipation | 11 | 3.96 | 4 | 4.30 | 0 | 0.00 | 7 | 6.93 | 0.054 |
| Dizziness | 11 | 3.96 | 3 | 3.23 | 2 | 2.38 | 6 | 5.94 | 0.422 |
| Fatigue | 13 | 4.68 | 7 | 7.53 | 1 | 1.19 | 5 | 4.95 | 0.135 |
| Headache | 40 | 14.39 | 12 | 12.90 | 16 | 19.05 | 12 | 11.88 | 0.339 |
| Insomnia | 12 | 4.32 | 5 | 5.38 | 1 | 1.19 | 6 | 5.94 | 0.236 |
| Nausea | 34 | 12.23 | 14 | 15.05 | 6 | 7.14 | 14 | 13.86 | 0.227 |
| Somnolence | 11 | 3.96 | 5 | 5.38 | 1 | 1.19 | 5 | 4.95 | 0.294 |

*AE: adverse events, Ari: aripiprazole, rTMS: repetitive transcranial magnetic stimulation, Ven/ Dul: venlafaxine duloxetine*

**Supplementary Table 4:** Mean Number of Treatment Emergent AEs and SAEs per Participant by Treatment Group (Mean, STD)

|  | **All Participants**  **(n=278)** | **Aripiprazole Group**  **(n=93)** | **rTMS Group**  **(n=84)** | **Venlafaxine**  **Duloxetine**  **Group**  **(n=101)** | **p-value** |
| --- | --- | --- | --- | --- | --- |
| AEs | 1.39 (2.0) | 1.62 (1.9) | 1.01 (1.7) | 1.50 (2.3) | 0.103 |
| SAEs | 0.011 (0.10) | 0.0 (0) | 0.012 (0.11) | 0.020 (0.14) | 0.411 |

*AE: adverse events, SAE: serious adverse events, rTMS: repetitive transcranial magnetic stimulation.*

**Supplementary Table 5:** Mean Number of Treatment Emergent AEs for each Specific AE affecting 3% or more of Participants.

| **Adverse Event** | **All Participants Mean** | **All Participants SD** | **Ari**  **Group Mean** | **Ari Group SD** | **rTMS Group Mean** | **rTMS Group SD** | **Ven/ Dul Group Mean** | **Ven/ Dul Group SD** | **p-value** |
| --- | --- | --- | --- | --- | --- | --- | --- | --- | --- |
| Constipation | *0.04* | *0.2* | *0.04* | *0.2* | *0.00* | *0.0* | *0.07* | *0.3* | *0.054* |
| Dizziness | *0.04* | *0.2* | *0.03* | *0.2* | *0.02* | *0.2* | *0.06* | *0.2* | *0.425* |
| Fatigue | *0.05* | *0.3* | *0.09* | *0.3* | *0.01* | *0.1* | *0.06* | *0.3* | *0.152* |
| Headache | *0.19* | *0.6* | *0.17* | *0.5* | *0.29* | *0.7* | *0.14* | *0.4* | *0.180* |
| Insomnia | *0.04* | *0.2* | *0.05* | *0.2* | *0.01* | *0.1* | *0.06* | *0.2* | *0.238* |
| Nausea | *0.14* | *0.4* | *0.17* | *0.4* | *0.12* | *0.5* | *0.14* | *0.3* | *0.703* |
| Somnolence | *0.04* | *0.2* | *0.06* | *0.3* | *0.01* | *0.1* | *0.05* | *0.2* | *0.268* |

*AE: adverse events, Ari: aripiprazole, rTMS: repetitive transcranial magnetic stimulation, Ven/ Dul: venlafaxine duloxetine*

**Supplementary Table 6*:*** Highest Level of Severity of AEs by Group among those with a Treatment Emergent AE (N, %).

|  | **All Participants**  **(n=136)** | **Aripiprazole Group**  **(n=53)** | **rTMS Group**  **(n=33)** | **Venlafaxine**  **Duloxetine**  **Group**  **(n=50)** | **p-value** |
| --- | --- | --- | --- | --- | --- |
| Mild | 60 44.1% | 25 47.2% | 13 39.4% | 22 44.0% | 0.458 |
| Moderate | 69 50.7% | 25 47.2% | 20 60.6% | 24 48.0% |  |
| Severe | 7 5.2% | 3 5.7% | 0 0.0% | 4 8.0% |  |

*AE: adverse events*

**Supplementary Table 7**: Sensitivity Analysis - Primary analysis excluding participants randomized to Venlafaxine/ Duloxetine (N=20) while rTMS randomizations were paused due to COVID

| Outcome | Treatment Groups | Mean level (SD) | Mean slope (SD) | P-value  Treatment group main effect | p-value  Treatment group by time interaction effect |
| --- | --- | --- | --- | --- | --- |
| MADRS | rTMS | -17.38 (1.2) | -8.93 (1.1) | 0.241 | 0.015 |
| MADRS | Venlafaxine/ Duloxetine | -13.12 (1.2) | -6.22 (0.8) |  |  |

*rTMS: repetitive transcranial magnetic stimulation,* SD: Standard deviation, MADRS: Montgomery Asberg Depression Rating Scale,
